# First-Principles Study on the Nanofriction Properties of Diamane: The Thinnest Diamond Film

**DOI:** 10.3390/nano12172939

**Published:** 2022-08-26

**Authors:** Jianjun Wang, Lin Li, Jiudong Wang, Wentao Yang, Peng Guo, Meng Li, Dandan Liu, Haoxian Zeng, Bin Zhao

**Affiliations:** 1 Zhengzhou Key Laboratory of Low-Dimensional Quantum Materials and Devices, College of Science, Zhongyuan University of Technology, Zhengzhou 450007, China; 2 Delivery & Devices Research and Development, Eli Lilly and Company, Indianapolis, IN 46285, USA

**Keywords:** diamane, friction, charge distribution, density functional theory (DFT)

## Abstract

Diamane, the thinnest sp^3^-hybridized diamond film, has attracted great interest due to its excellent mechanical, electronic, and thermal properties inherited from both graphene and diamond. In this study, the friction properties of surface hydrogenated and fluorinated diamane (H- and F-diamane) are investigated with dispersion-corrected density functional theory (DFT) calculations for the first time. Our calculations show that the F-diamane exhibits approximately equal friction to graphene, despite the presence of morphological corrugation induced by sp^3^ hybridization. Comparative studies have found that the coefficient of friction of H-diamane is about twice that of F-diamane, although they have the same surface geometric folds. These results are attributed to the packed charge surface of F-diamane, which can not only effectively shield carbon interactions from two contacting films, but also provide strong electron–electron repulsive interaction, resulting in a large interlayer distance and a small wrinkle of potential energy at the interface. The interesting results obtained in this study have enriched our understanding of the tribological properties of diamane, and are the tribological basis for the design and application of diamane in nanodevices.

## 1. Introduction

Carbon (C) can form different kinds of nanostructures, from fullerene to nanotubes to graphene, which are milestones in the development of nanomaterials and have attracted immense research activities [1,2]. Recently, diamane, the thinnest sp^3^-hybridized diamond film, has attracted much attention due to its outstanding electronic, mechanical, and thermal properties inherited from graphene and diamond [3,4,5]. In 2009, Chernozatonskii et al., for the first time, theoretically predicted the diamane properties based on the density functional theory (DFT) calculations, which opens a band gap and exhibits higher stiffness compared to graphene. Motivated by the theoretical predictions, several groups have been trying to convert sp^2^-hybridized bilayer graphene into ultra-thin sp^3^-hybridized diamane. Several theoretical and experimental studies show that although the conversion of sp^2^ to sp^3^ can be achieved by applying high pressure, release of the pressure causes the return to the bilayer graphene, which indicates that diamane with pristine surfaces makes little sense [6,7,8,9,10]. Surface atom passivation is considered as an effective method to suppress surface activity and stabilize surface structure. It was indeed predicted that hydrogen (H) and fluorination (F) passivated diamane (H-diamane, F-diamane) films have sufficient stability due to the larger binding energy between C and passivation atoms and the higher thermal stability [11]. Very recently, H-diamane and F-diamane were prepared experimentally by different research groups [12,13,14]. In H-diamane (F-diamane), two C atoms of two subatomic lattices with H (F) atoms form a layered unit, while the other non-attached C atoms form a covalent bond to the sub-lattices of other C atoms of neighboring graphene, thereby leading to the formation of a new carbon system [4].

As the thinnest diamond film, diamane exhibits excellent physical and chemical characteristics. DFT calculations have revealed that diamane has a better band gap than graphene, and this band gap can be further tuned over a wide range by using doping [15], an external electrical field [4], and surface functionalization [3,11,12,16,17]. Cheng et al. showed that diamane has lower effective masses of conduction electrons than those in bulk diamond, making it an alternative material for electronic applications [18]. Parallel to the electronic structure, the mechanical properties of diamane are of particular interest. Inherited from its bulk counterpart, theoretical simulations show that diamane possesses much higher stiffness and elastic constants than that of graphene [19]. Experiments also found that the indentation modulus and bending stiffness of diamane are comparable to diamond, and much higher than graphene [10,20]. Besides the high stiffness, diamane displays exceptional thermal properties. It was predicted that diamane has a giant thermal conductivity of 2240 Wm^−1^K^−1^ at 300 K, which is higher than most two-dimensional (2D) materials [21]. Such superior electronic, mechanical, and thermal properties suggest that diamane may be a good candidate in micro- and nanoelectromechanical systems (MEMS/NEMS) and the next generation of nanodevices.

The increase in the surface-to-volume ratio that occurs when a device is scaled down in size makes friction increasingly problematic in nanodevices. Therefore, it is necessary to understand the friction properties of diamane for its application in MEMS/NEMS. After inheriting excellent lubricant properties of its bulk graphite, graphene has been considered as one of the most promising nanoscale lubricating materials [2,22], which can be used as an excellent coating lubricant to reduce interface friction and wear [23,24]. Diamond single crystal (DSC) film also has low friction and high wear resistance properties [25], which can be understand by the mechanisms of the sliding-induced graphitization [26,27] and passivation of dangling bonds [28,29]. Although the frictional properties of graphene and DSC are well understood, it is not easy to infer the frictional properties of diamane, because the nanoscale frictional properties are decided by many factors, such as interface environment [30,31], commensurability [32,33], and size effect [34]. As in the statement above, although the mechanical, electronic, and thermal properties of diamane have been widely discussed, to our knowledge, the friction properties of diamane have not yet been reported, despite the fact that it is a critical parameter to consider in the use of diamane in nanodevices. Therefore, comprehensive studies on the friction properties of diamane are urgently needed.

In this study, based on the DFT calculations, we investigate the interlayer friction properties of the bilayer H-diamane and bilayer F-diamane. An interesting finding is that the sp^3^-hybridized F-diamane exhibits a coefficient of friction (*μ*) similar to sp^2^-hybridized graphene, and the *μ* can be further tuned by choosing passivation atoms. This study provides quantitative friction information for diamane, which is the tribological basis for its design and application in nanodevices.

## 2. Methods

In this study, we used the Vienna Ab Initio Simulation Package (VASP) code to carry out DFT calculations [35,36,37]. The project augmented wave (PAW) method was used to treat the electron–ion interactions [35,36]. The exchange–correlation interactions were handled with the generalized gradient approximation (GGA) in PBE [38]. The van der Waals (vdW) interactions were added by the DFT-D2 approach, with a scaling factor *S*_6_ = 0.75 [39]. We chose an energy cutoff of 600 eV and 21 × 21 × 21 Monkhorst–Pack grid for 2D irreducible Brillouin zone integration [40]. The convergence thresholds of total energy and the Hellmann–Feynman force were set to 10^−5^ eV and 0.01 eV/Å, respectively. The convergence thresholds used in this paper were tested, as shown in the Figure S1 of Supporting Information. We set up a vacuum layer of about 20 Å to avoid the interactions between two adjacent cells. The friction properties of the diamane were separately calculated by the potential energy surface (PES) method and the maximum energy barrier method [41,42]. The Device Studio program was used for visualization and modeling [43].

## 3. Results and Discussion

**Figure 1:**
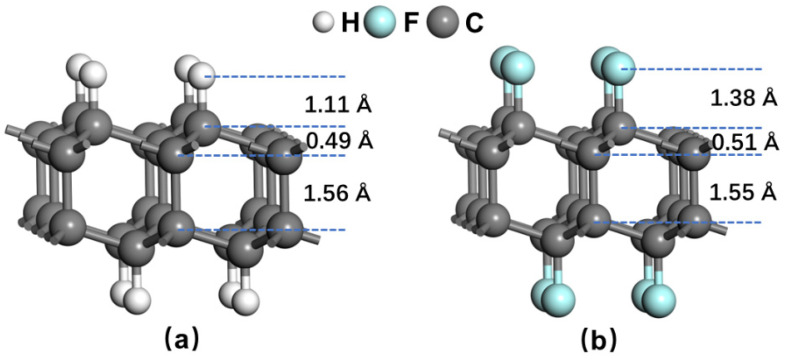
The optimized geometry structures of (**a**) H-diamane and (**b**) F-diamane.

We first optimized the structures of the diamane, as shown in Figure 1. The calculated in-plane lattice constants are 2.52 and 2.56 Å for H-diamane and F-diamane, respectively, which are very close to other computational and experimental results [11,14,16,17,44]. The two systems exhibit almost identical interlayer C-C bond lengths of 1.56 Å, which is very close to the value of bulk diamond. Due to the interactions from passivated atoms and the carbon atoms in the adjacent layers, a geometric fold of 0.5 Å appears in the graphene, which may cause additional friction following the atomic roughness theory of friction [45]. It should be noted that C-H bond length in H-diamane is shorter than the C-F bond length in F-diamane, which can be attributed to the much greater charge and larger electronegativity of F than H.

**Figure 2:**
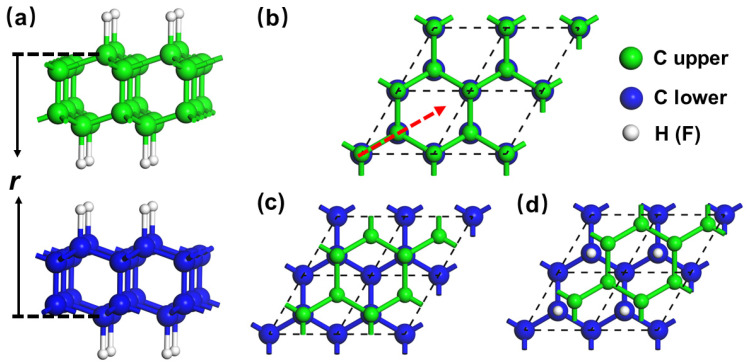
Sliding model. (**a**,**b**) Represent side and top views of the initial configuration, respectively. The vertical distance between the bottom and top C layers is defined as *r*. As all interfacial C and H atoms from two layers face each other, the configuration is defined as top stacking. (**c**,**d**) are defined as hollow and bridge stackings following previous study [49]. To clearly exhibit the stacking characters, the C atoms in different sheets are labeled with different colors.

Based on the optimized structures, two sheets of H-diamane (H-diam/H-diam) or F-diamane (F-diam/F-diam) were placed to slide against each other along a path to model the friction process, as shown in Figure 2. As the upper layer of diamane slides across the lower one along the diagonal of two primitive lattice vectors (the red dotted arrow in Figure 2b), the highly symmetric top (Figure 2b), hollow (Figure 2c), and bridge (Figure 2d) stackings will alternately appear on the path. According to our previous studies [30,46,47,48], the friction properties of the hexagonal system are decided by these three stacks.

As friction properties are closely related to the interfacial interactions, we firstly calculated the interaction energy (*E*_IE_) of the sliding system. *E*_IE_ was calculated by using the formula, $E_{\mathrm{IE}}=E^{\mathrm{AB}}(r)-E^{A}-E^{B}$, where *E*^AB^(*r*) is the total energy of the two contacting films at the distance of *r*, and *E*^A^ (*E*^B^) is the energy of the separate film. For each interlayer distance *r*, only the C atoms in the bottom layer of the lower slab and the topmost layer of the upper slab were kept frozen, whereas all other atoms were relaxed in all our calculations. Therefore, the vertical distance between the two fixed C layers was defined as *r*, as shown in Figure 2a. The load effect was applied by setting *r* values [29,46]. According to the definition of *E*_IE_, a more negative *E*_IE_ indicates better stability.

**Figure 3:**
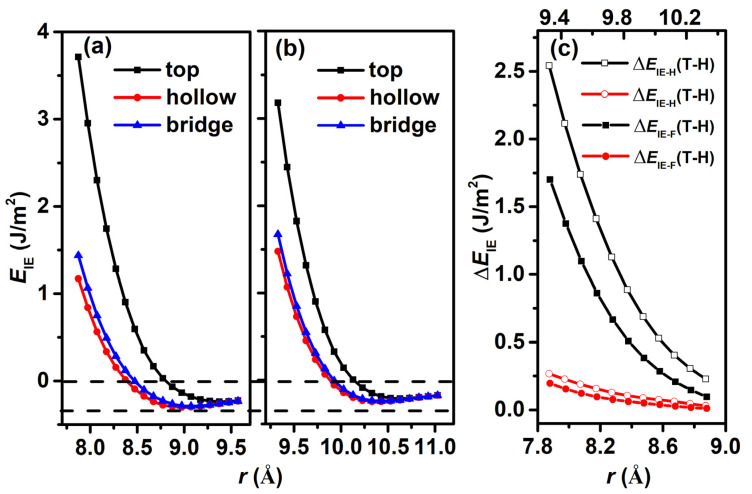
Interaction energy *E*_IE_ as a function of interlayer space *r* between two sheets of (**a**) H-diam/H-diam and (**b**) F-diam/F-diam, respectively. (**c**) The difference in *E*_IE_ between top and hollow stackings (Δ*E*_IE__-H_(T-H) for H-diam/H-diam, Δ*E*_IE-F_(T-H) for F-diam/F-diam), and between bridge and hollow stackings (Δ*E*_IE-H_(B-H) for H-diam/H-diam, Δ*E*_IE-F_(B-H) for F-diam/F-diam) as a function of *r* in both systems.

The calculated *E*_IE_ values as a function of *r* for top, bridge, and hollow stackings are shown in Figure 3a,b for H-diam/H-diam and F-diam/F-diam, respectively. Comparisons of *E*_IE_ in different stackings reveal that both systems have the strongest interaction energy at the top stacking, followed by the bridge one, and hollow stacking has the weakest values. This behavior occurs because interfacial C, H, and F atoms exhibit large spaces for movement and avoid repulsive forces at both hollow and bridge stackings. To reveal the difference in interlayer binding between the two systems, we compared the *E*_IE_ for the two systems. The first striking difference between the two systems is that the F-diam/F-diam system has a larger equilibrium interlayer distance (the interlayer distance corresponding to the lowest *E*_IE_) of about 10 Å; for the H-diam/H-diam system, it is about 9 Å. The binding energy (the absolute value of lowest *E*_IE_) of F-diam/F-diam systems is smaller than that of the H-diam/H-diam system, which means that the interlayer repulsive interaction is stronger in the F-diam/F-diam system. The second interesting difference is that the expansion window widths of the three lines in the H-diam/H-diam are larger than those in the F-diam/F-diam system, which indicates that H-diam/H-diam has a larger difference in *E*_IE_ among the three stackings. To clearly exhibit these differences, we further calculated the difference in *E*_IE_ between different stackings for both systems. Figure 3c clearly shows that the Δ*E*_IE-H_(T-H) is larger than that of Δ*E*_IE-F_(T-H), and Δ*E*_IE-H_(B-H) is larger than Δ*E*_IE-F_(B-H), which indicates that the small friction in the F-diam/F-diam system can be expected in the view of *E*_IE_ and interlayer distance *r*.

The variation of the *E*_IE_ as a function of the relative lateral position of the two contacting films at their equilibrium interlayer distance *z*_eq_, ∆*E*_IE_ (x, y, *z*_eq_), is defined as the PES [41], from which one can obtain the overall friction characteristics of a contacting interface at a zero normal load. The corrugation of the PES determines the intrinsic resistance to sliding, which is also the maximum energy that can be dissipated during sliding processes.

**Figure 4:**
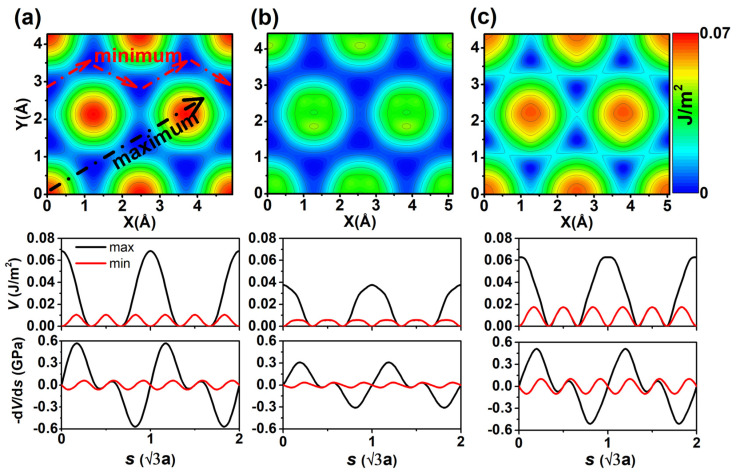
Potential energy surfaces (PES). (**a**) H-diam/H-diam, (**b**) F-diam/F-diam, and (**c**) graphene-graphene systems. The corresponding potential barrier *V* and lateral stress τ=−dV/ds as functions of sliding distance s (in unit of √3 times of lattice constant) along the minimum and maximum barrier paths are plotted under each PES. The minimum is taken as a reference.

The calculated PESs for H-diam/H-diam and F-diam/F-diam are shown in Figure 4a,b, respectively. Figure 4c shows the PES of bilayer graphene (graphene–graphene), which can be used as a benchmark for the first two systems. From Figure 4, we can see that the three systems exhibit similar shapes of PES, with the largest and smallest barriers in top and hollow stackings, respectively. The obvious difference between Figure 4a,b is that H-diam/H-diam has a larger PES corrugation of about 0.07 J/m^2^, which is almost twice that of the F-diam/F-diam system. The sliding barriers *V* and the lateral stress τ=−dV/ds along two paths (bottom plane of Figure 4) in H-diam/H-diam are twice those of the F-diam/F-diam system, which is consistent with the previous prediction that F-diam/F-diam has a smaller friction than that of the H-diam/H-diam system. It should be noted that the *V* and *τ* of graphene–graphene are larger than those of F-diam/F-diam, and are slightly smaller than those of H-diam/H-diam, which indicates that the diamane system keeps the excellent friction properties of graphene, and the conversion of sp^2^ to sp^3^ does not change the friction properties of graphene.

**Figure 5:**
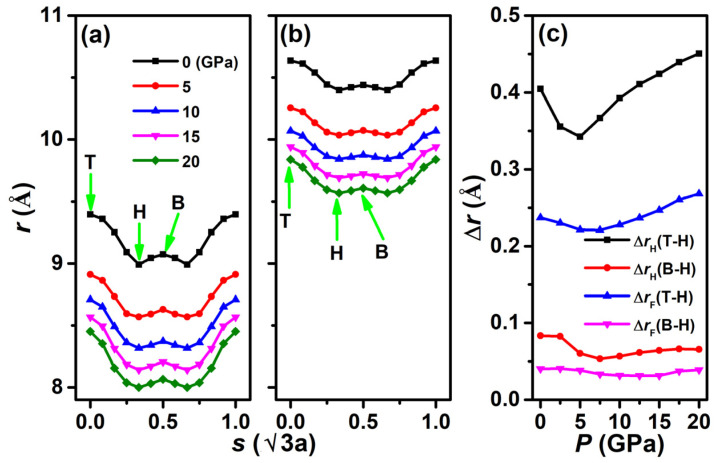
Interlayer distances *r* as a function of sliding distance *s* (in unit of √3 times of lattice constant, *s* = 0, 0.33, and 0.5 represent top (T), hollow (H), and bridge (B) stackings, respectively) along the maximum path (see Figure 4a) under different normal pressures P. (**a**) H-diam/H-diam and (**b**) F-diam/F-diam systems. (**c**) The difference in *r* between top and hollow (Δ*r*_H_(T-H) for H-diamane, Δ*r*_F_(T-H) for F-diamane), bridge, and hollow stackings (Δ*r*_H_(B-H) for H-diamane, Δ*r*_F_(B-H) for F-diamane) as a function of *P* for both systems.

The friction properties calculated by the PES method do not consider a normal load. In the following section, we will consider the load effect on friction. One normal load can be applied in our systems by setting a certain *r* according to the formula $F_{N}=-dE_{IE}/dr$ [42]. Figure 5 shows the relationship between *F*_N_ and *r* along the maximum path (show in Figure 4a) under normal pressures of 0~20 GPa. The two systems exhibit the same characteristics that all curves exhibit their maximum and minimum at the top and hollow configurations, and the interlayer distance *r* decreases with the increase in *F*_N_. The most obvious difference is that the *r* in the F-diam/F-diam (Figure 5b) is much higher than that of the H-diam/H-diam system (Figure 5b) under the same load, which is consistent with the results in Figure 2. The relative fluctuation height of the interlayer distance curves along the sliding path were calculated, as shown in Figure 5c. From Figure 5c, we can clearly see that H-diam/H-diam has a larger relative fluctuation height (Δ*r*_H_(T-H) and Δ*r*_H_(B-H)) than that of the F-diam/F-diam (Δ*r*_F_(T-H) and Δ*r*_F_(B-H)) system, and the difference increases with an increase in pressure. The fluctuations are determined by the differences in interlayer interactions for different configurations along the sliding direction. Therefore, we can infer that the friction in H-diam/H-diam is still larger than that of F-diam/F-diam under the same pressure.

**Figure 6:**
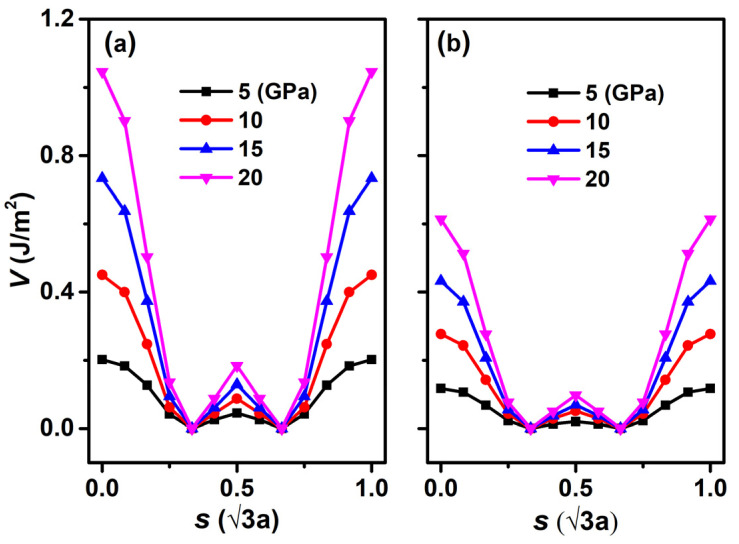
Potential energy *V* curves as a function of sliding distance *s* (in unit of √3 times of lattice constant) under different loads. (**a**) H-diam/H-diam and (**b**) F-diam/F-diam.

According to the method of Zhong et al. [42], potential energy under the load of *F*_N_ can be calculated using the formula

(1) $$V\left(s,F_{N}\right)=E_{IE}\left(s,r\left(s,F_{N}\right)\right)+F_{N}r\left(s,F_{N}\right)-V_{0}\left(f_{N}\right)$$

the potential energy *V*(*s*, *F*_N_) includes two components: one is the variation of the *E*_IE_ under load, and the other is the work against the external force *F*_N_ applied to the system, with *V*_0_(*F*_N_) as the minimum in the sliding path. Thus, *V*(*s*, *F*_N_) is the relative potential barrier along the sliding path. The potential energy curves under different loads are shown in Figure 6. Similar to the curves of interlayer distance *r* in Figure 5, all of the potential energy curves for both systems exhibit the maximum and minimum at the top and hollow configurations, and the *V*(*s*, *F*_N_) increases with an increase in *F*_N_. The most obvious difference is that the *V*(*s*, *F*_N_) in the H-diam/H-diam system (Figure 6a) is much larger than that of F-diam/F-diam (Figure 6b) under the same load.

**Figure 7:**
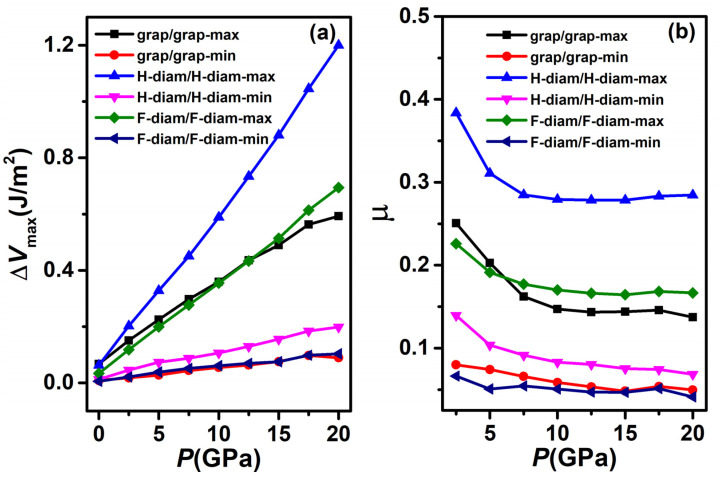
(**a**) Potential energy barrier Δ*V*_max_ and (**b**) coefficient of friction *μ* along both of minimum and maximum barrier paths under different loads *P* for graphene–graphene, H-diam/H-diam, and (**b**) F-diam/F-diam systems.

The coefficient of friction *μ* is calculated by the following formula

(2) $$\mu =\Delta V_{\max}/\left(\Delta s\cdot F_{N}\right)=\left(V_{\max}\left(F_{N}\right)-V_{\min}\left(F_{N}\right)\right)/\left(\Delta s\cdot F_{N}\right)$$

where Δ*V*_max_ is the potential energy difference between the maximum and minimum along the sliding path under the load of *F*_N_, and Δ*s* is the distance between the positions of maximum and minimum potential energies. The Δ*V*_max_ and *μ* along the maximum and minimum paths (shown in Figure 4a) for H-diam/H-diam, F-diam/F-diam, and graphene–graphene systems are shown in Figure 7. For the Δ*V*_max_ (Figure 7a), all the curves of Δ*V*_max_ increase with an increase in the normal load, and the slope of the barrier curve of the maximum path is greater than that of the minimum path, indicating that the sliding barrier on the maximum path is more sensitive to external load. Comparing the three systems shows that graphene–graphene and F-diam/F-diam systems have almost the same Δ*V*_max_ for both paths, which is smaller than that of H-diam/H-diam. This result is also reflected by the *μ* in Figure 7b. Figure 7b shows that graphene–graphene and F-diam/F-diam systems exhibit almost the same *μ*, which is about one half of that of the H-diam/H-diam system for both minimum and maximum paths. The comparisons of friction indicate that sp^3^-hybridized diamane can inherit excellent friction properties from graphene through atom surface passivation, and the friction is closely related to the species of passivated atom. It should be noted that during friction in the non-adiabatic regime, the sliding is fast enough that the system does not relax the internal stress due to the lateral strain caused by the sliding. However, in this study, the atoms are allowed to relax when calculating the PESs, so the effect of elastic deformation on friction is ignored.

**Figure 8:**
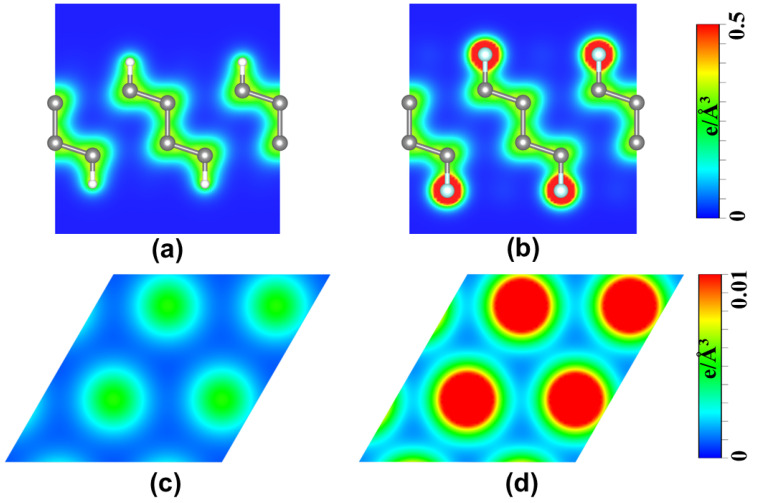
Maps of the charge density. Profile charge density plots along the maximum path of (**a**) H-diamane and (**b**) F-diamane. Charge distribution at a distance of 1 Å from the top passivated atoms layer for (**c**) H-diamane and (**d**) F-diamane.

It is well known that the friction at atom scale is fundamentally determined by the charge distribution [30,46,47,50]. To understand the friction difference between H-diam/H-diam and F-diam/F-diam systems, the charge distribution of the H-diamane and F-diamane structures were calculated, as shown in Figure 8. The cross-sectional view of charge density along the maximum path (shown in Figure 4a) shows that the interfacial charge in F-diamane (Figure 8a) is much higher than that of H-diamane (Figure 8b). This can be understood in two aspects. First, there are one and seven valence electrons in H and F atoms, respectively, and thus the intrinsic charge in the surface of F-diamane is larger than that of H-diamane. Moreover, the larger electronegativity difference between F and C in F-diamane causes the charge to accumulate near the F atom at the surface, but the charge in H tends to transfer to the middle of C-H in H-diamane, due to the similar electronegativity of C and H. To confirm this, the charge distribution at a distance of 1 Å from the top layer was calculated, which clearly shows that the charge almost covered the entire surface of F-diamane (Figure 8d) compared with H-diamane (Figure 8c). The packed charge surface of F-diam/F-diam can not only effectively shield C interactions from two contacting films, but also provide stronger electron–electron repulsive interactions, resulting in a large interlayer distance and a small wrinkle of potential barrier at the interface [29,51]. Consequently, the *μ* of the F-diam/F-diam is smaller than that of the H-diam/H-diam system.

Several research groups have studied the effect of passivation atoms on the friction properties of graphene and diamond films, and obtained different friction behavior and mechanisms. For flat sp^2^-hybridized graphene, the surface passivation usually causes an increase in friction. Based on the friction force microscopy (AFM) measurements, Ko et al. showed that hydrogenated, fluorinated, and oxidized graphenes exhibit, two-, six-, and seven-fold enhanced nanoscale friction on their surfaces, respectively, compared to pristine graphene [52]. They attributed the increase in friction to an increase in out-of-plane elasticity. In AFM experiments, Li et al. observed that the friction between a silicon atomic tip and fluorinated graphene can range from 5−9 times higher than for graphene, and they attributed the increase in friction to the increased potential energy corrugation caused by fluorine atoms [53]. Our DFT calculations show that the interlayer friction between two sheets of hydrogenated graphene is about twice that of bilayer graphene due to the increased roughness of the surface charge distribution [30]. However, for diamond films, atom passivation usually reduces interfacial friction [46,54,55]. The main mechanism is that the passivation atoms can shield the chemical interactions between the relative sliding surfaces. Obviously, the shielding mechanism is still valid in the H- and F-diamane systems.

Finally, we discuss the influence of the thickness of diamond film on its tribological properties. The friction properties of hydrogenated and fluorinated diamond films from 3 to 8 layers were calculated. It is found that thickness has a negligible influence on the friction, and all the films, from the thinnest diamane to the eight-layer diamond film (which can be used to simulate bulk diamond film [56]) exhibit approximately the same *μ* (see Figure S2 in Supporting Information). This phenomenon can be attributed to the strong sp^3^ bond in the diamond film, which makes the structure less prone to deformation under pressure. Different from diamond films, previous studies have shown that the friction of graphene decreases with an increase in thickness due to the weak vdW interlayer interaction [34]. The more layers of graphene, the smaller the out-of-plane deformation under the external force, and the smaller the friction.

## 4. Conclusions

The friction properties of diamane were studied by the first-principles method based on DFT. It was found that F-diamane with geometric folds in the carbon layer has roughly the same *μ* as flat graphene because the folds can be shielded by the packed charges of the passivated fluoride layer. We further compared the frictional properties of H-diamane/H-diamane and F-diamane/F-diamane, and found that the *μ* of former system is about twice as large as the latter, which can be attributed to the electronic structure difference between the H-diamane and F-diamane. The charge density distribution shows that the charge surface of F-diamane is more packed than that of H-diamane, which can not only effectively block the interaction between two carbon films, but also enhance electron–electron repulsion between the two F layers. Thus F-diamane/F-diamane exhibits a larger interlayer distance and a smaller binding energy and friction, indicating that the frictional properties of diamane are closely related to the electronic structure of passivated atoms. This study shows that in addition to the high hardness, good band gap, and excellent heat conduction properties reported in previous studies, the thinnest sp^3^-hybridized diamond films also have excellent friction properties comparable to graphene, and the friction properties can be modulated by changing passivated atoms. The results enrich the knowledge of diamane in tribology and provide a tribological basis for the wide application of diamane in micro- and nano-devices.

## Data Availability

The data presented in this study are available on request from the corresponding authors.

## Supplementary Materials

The following supporting information can be downloaded at: https://www.mdpi.com/article/10.3390/nano12172939/s1, Figure S1: Interaction energies (EIE) under different convergence thresholds for top (top) and hollow (hol) stackings; The difference of interaction energies (ΔEIE) between two convergence thresholds for both top and hollow stackings. Figure S2: (a) Potential energy barrier ΔVmax and (b) coefficient of friction μ along both of minimum and maximum paths as a function of diamond film thickness under normal pressure of 10 GPa.
