# Supplementary material for: First-Principles Study on the Nanofriction Properties of Diamane: The Thinnest Diamond Film
Source: Nanomaterials (Basel). 2022 Aug 26;12(17):2939. doi: 10.3390/nano12172939 (PMC9457850; doi:10.3390/nano12172939)
Supplement: Supplementary file 1 [file nanomaterials-12-02939-s001.zip › nanomaterials-1865205-supplementary.pdf]

Supporting Information

First-Principles Study on the Nanofriction Properties of Diamane: The Thinnest Diamond Film

Jianjun Wang [1,*], Lin Li [2], Jiudong Wang [1], Wentao Yang [1], Peng Guo [1], Meng Li [1], Dandan Liu [1], Haoxian Zeng [1] and Bin Zhao [1]

[1] Zhengzhou Key Laboratory of Low-Dimensional Quantum Materials and Devices, and College of Science, Zhongyuan University of Technology, Zhengzhou 450007, China

[2] Delivery & Devices Research and Development, Eli Lilly and Company, Indianapolis, IN 46285, USA

1. The POSCAR files of geometries for the optimized H-diamane and F-diamane systems

(a) POSCAR file of H-diamane

```
H-diamane
   1.00000000000000
     2.1823840175256000    -1.2600000000000000     0.0000000000000000
     0.0000000000000000     2.5200000000000000     0.0000000000000000
     0.0000000000000000     0.0000000000000000    40.0000000000000000
   C    H
     4     2
Selective dynamics
Direct
  0.3333333333333334  0.6666666666666667  0.1635133302329016   T   T   T
  0.0000000000000000  0.0000000000000000  0.1513013220826170   T   T   T
  0.0000000000000000  0.0000000000000000  0.1122978985994153   T   T   T
  0.6666666666666667  0.3333333333333334  0.1000000000000000   F   F   F
  0.6666666666666667  0.3333333333333334  0.0723612503567072   T   T   T
  0.3333333333333334  0.6666666666666667  0.1912339698994699   T   T   T
```

(b) POSCAR file of F-diamane

```
F-diamane
   1.00000000000000
     2.2170250336768000    -1.2800000000000000     0.0000000000000000
     0.0000000000000000     2.5600000000000000     0.0000000000000000
     0.0000000000000000     0.0000000000000000    40.0000000000000000
   C    F
     4     2
Selective dynamics
Direct
  0.3333333333333334  0.6666666666666667  0.1640131393999717   T   T   T
  0.0000000000000000  0.0000000000000000  0.1514255418435193   T   T   T
  0.0000000000000000  0.0000000000000000  0.1125890375472634   T   T   T
  0.6666666666666667  0.3333333333333334  0.1000000000000000   F   F   F
  0.6666666666666667  0.3333333333333334  0.0655595363652450   T   T   T
  0.3333333333333334  0.6666666666666667  0.1984531207438430   T   T   T
```

2. Figures

Figure S1

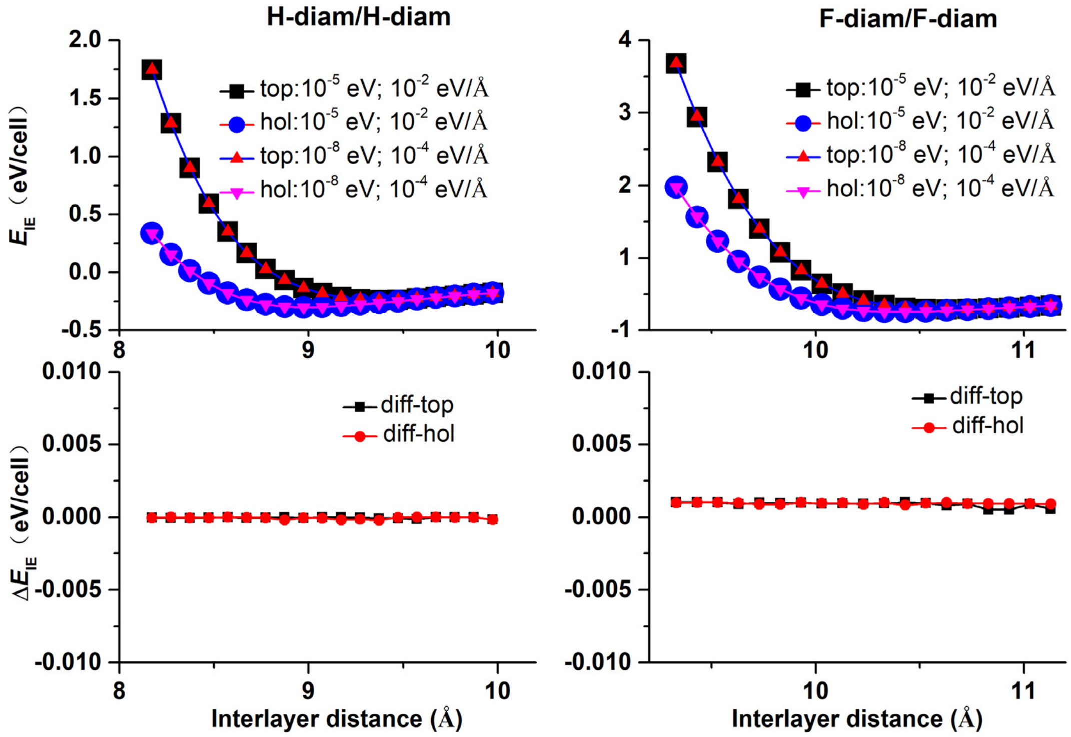

Figure S1. Interaction energies (E_{IE}) under different convergence thresholds for top (top) and hollow (hol) stackings; The difference of interaction energies (ΔE_{IE}) between two convergence thresholds for both top and hollow stackings.

The convergence thresholds used in this paper have been tested. In the Figure S1, we exhibit the interaction energies (E_{IE}) of the two systems at the maximum (top) and minimum (hollow) energy stackings under two different convergence thresholds (10^{-5} eV and 0.01 eV/Å; 10^{-8} eV and 10^{-4} eV/Å). It can be seen from comparison that the difference of E_{IE} between the two convergence thresholds are almost negligible. We further compared the difference of interaction energies (ΔE_{IE}) between two convergence thresholds for both top and hollow stackings, and found that the difference were within 0.0005 eV/atom. □s the friction is mainly determined by the E_{IE}, and therefore, our parameter selection is reliable for the calculation of friction considering the computational efficiency.

Figure S2

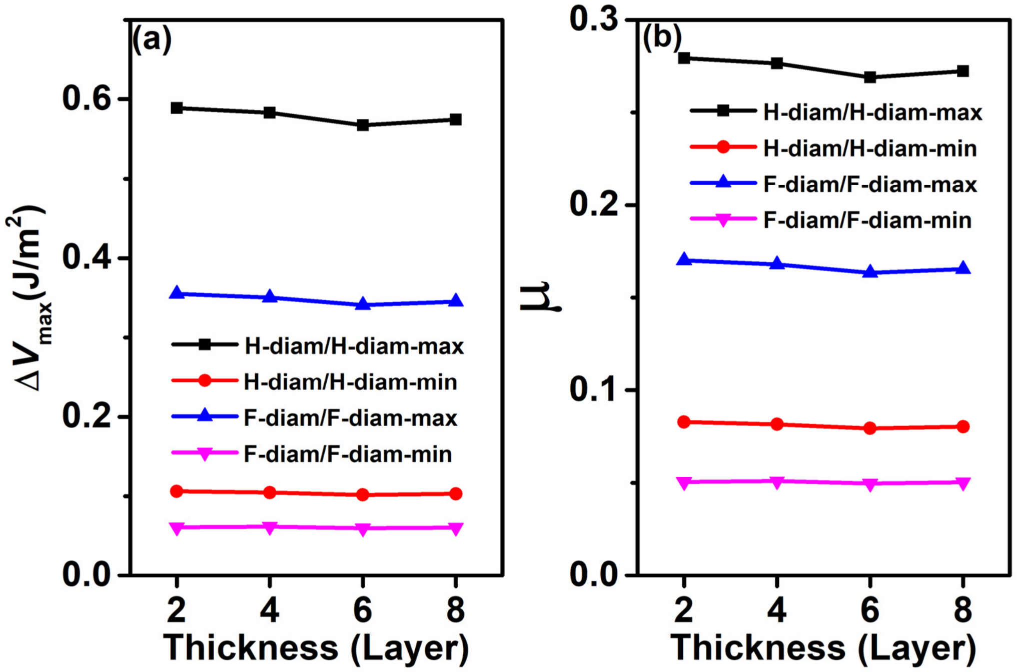

Figure S2. (a) Potential energy barrier ΔV_{max} and (b) coefficient of friction μ along both of minimum and maximum paths as a function of diamond film thickness under normal pressure of 10 GPa.

We have compared the sliding barrier and COFs of diamond films with thickness of 2~8 layers under the pressure of 0~20GPa. It is found that with the increase of thickness, the changes of sliding barrier and COF of the two systems are almost negligible for both maximum and minimum paths, that is, the friction does not change with thickness. The case at 10GPa is exhibited in the Figure S2.
